# Increased Th17/Treg Ratio in Poststroke Fatigue

**DOI:** 10.1155/2015/931398

**Published:** 2015-06-18

**Authors:** Xinjing Liu, Komal Kenkare, Shanshan Li, Varsha Desai, John Wong, Xun Luo, Lisa J. Wood, Yuming Xu, Qing Mei Wang

**Affiliations:** ^1^Neurology Department, The First Affiliated Hospital of Zhengzhou University, 1 East Jianshe Road, Zhengzhou, Henan 450052, China; ^2^School of Nursing, Fatigue Research Laboratory, MGH Institute of Health Professions, 36 1st Avenue, Charlestown, MA 02129, USA; ^3^Basic Medical College, Guangdong Pharmaceutical University, Guangzhou 510006, China; ^4^Spaulding Rehabilitation Hospital, 300 First Avenue, Charlestown, MA 02129, USA; ^5^School of Nursing, Fatigue Research Laboratory, MGH Institute of Health Professions, 96 13th Street, Charlestown, MA 02129, USA; ^6^Department of Rehabilitation Medicine, Fourth Affiliated Hospital of Zhengzhou University, 169-10 Nanyang Road, Zhengzhou, Henan 450044, China; ^7^Stroke Biological Recovery Laboratory, Spaulding Rehabilitation Hospital, Harvard Medical School, 96 13th Street, Charlestown, MA 02129, USA

## Abstract

Fatigue is a major debilitating symptom after stroke. The biological mechanisms underlying poststroke fatigue (PFS) are unknown. We hypothesized that PSF is associated with an alteration in the balance between Th17 and Treg cells. To test this hypothesis we assessed fatigue in 30 stroke survivors using the Fatigue Scale for Motor and Cognitive Functions (FSMC). Peripheral blood was collected for assessment of Th17 and Treg cell populations and measurement of interleukin-10 (IL-10). Participants were dichotomized into severe fatigue (*n* = 14) and low-moderate fatigue (*n* = 16) groups by *K*-mean cluster analysis of FSMC scores. There were no group differences in age, gender, stroke type, stroke severity, or time since stroke. Stroke survivors in the severe fatigue group reported greater anxiety (*p* = 0.004) and depression (*p* = 0.001) than in the low-moderate fatigue group. The ratio of Th17 to Treg cells was significantly increased in the severe fatigue group relative to the mild-moderate fatigue group (*p* = 0.035). Serum levels of IL-10 negatively correlated withTh17/Treg ratio (*r* = −0.408,  *p* = 0.025). Our preliminary findings suggest that an imbalance in the Th17/Treg ratio is associated with the severity of PSF.

## 1. Introduction

Poststroke fatigue (PSF) is one of the most common symptoms following stroke with a prevalence ranging between 38% and 77% [1, 2]. PSF is characterized by difficulty in initiating or sustaining voluntary activities [3] and severely affects the ability to perform activity of daily living [4], reduces the ability to work [5], and increases overall mortality [6]. The biological mechanisms underlying poststroke fatigue (PFS) are unclear, and there is no effective treatment available. Although the cause of PSF remains unclear, several factors including depression, anxiety, and sleep disturbance have been shown to influence the severity of PSF. Immune-inflammatory alterations following stroke may contribute to PSF and these associated symptoms. This idea is based on the finding that fatigue is common in inflammatory and autoimmune disorders such as multiple sclerosis (MS), rheumatoid arthritis (RA), and systemic lupus erythematosus (LE) [7–9]. Brain inflammation is a key component of stroke related brain injury [10, 11]. Ischemic or hemorrhagic stroke leads to cellular injury which in turn stimulates an innate immune response [12].

Infiltrating cells secrete additional inflammatory molecules that further exacerbate brain injury. Two populations of infiltrating T helper cells, Th17 and regulatory T cells (Treg), originate from a common naïve T-cell precursor [13] and play important and opposing roles in regulating ischemic brain injury. IL-1*β* and IL-6 stimulate Th17 and Treg cell differentiation [14–16], and circulating levels of these cytokines in the acute phase after stroke predict the severity of PSF and poststroke depression (PSD) months thereafter. Th17 cells are characterized by their ability to produce the proinflammatory cytokines IL-17A and IL-17F [17]. Several studies in human trials suggest a pathogenic role of Th17 cells in inflammation and autoimmunity [18, 19]. Treg cells, a suppressive regulatory T helper cell subset, control autoreactive T-cell responses [20]. The anti-inflammatory effect of Treg cells is related in part to their secretion of IL-10, a potent anti-inflammatory cytokine [21]. An excess of proinflammatory Th17 cells relative to anti-inflammatory Treg cells has been implicated in chronic inflammation and autoimmunity [22, 23]. An imbalance in the ratio of Th17 to Treg cells has also been documented in stroke [24–26]. Li and coworkers reported an imbalance of Th17/Treg in patients with atherosclerotic cerebral infarction and also showed that the percentage of circulating Th17 cells was significantly elevated after stroke [25]. A similar increase in the percentage of Treg cells has also been found after stroke, but these cells possessed a reduced capacity to inhibit the proliferation of T cells [27]. Th17 cells have been implicated in the pathogenesis of chronic fatigue syndrome (CSF) [28–30]. Broderick and coworkers demonstrated that circulating levels of IL-6 and IL-23, which promote Th17 cell differentiation, were significantly increased in the CSF patients compared to controls [28].

Taken together, we hypothesized that an altered balance between Th17 and Treg may play a role in the development and/or perpetuation of PSF. In this pilot study we investigated the distribution of Th17 and Treg cells and serum levels of Treg-derived IL-10 in stroke patients to reveal a possible mechanism underlying PSF.

## 2. Subjects and Methods

### 2.1. Subjects

Thirty patients were recruited from the stroke rehabilitation outpatient clinic at Spaulding Rehabilitation Hospital. Inclusion criteria for subject enrollment included age of 18–90 years, clinical and radiographically confirmed ischemic or hemorrhagic cerebrovascular accident (CVA), and stroke onset > 3 months prior to study enrollment. Participants were ineligible to participate in the study if they had significant prestroke disability, decreased alertness, language reception, or attention that might interfere with understanding instructions to complete study questionnaires, excessive pain in any joint of the paretic extremity, advanced liver, kidney, cardiac, or pulmonary disease, terminal medical diagnosis consistent with survival <1 year, history of significant alcohol or drug abuse in the prior 3 years, active enrollement in a separate intervention study targeting stroke recovery, or current pregnancy. This study was approved by the Institutional Review Board, and all participants provided written informed consent.

### 2.2. Demographic and Clinical Data

Demographic and clinical data were collected, including age, gender, ethnicity, racial background, marital status, time since stroke (months), stroke type, stroke location, body mass index (BMI), smoking status, comorbidities, and treatment with immunosuppressant, steroids, or nonsteroid anti-inflammatory medication. Stroke severity was measured using modified Rankin Scale (mRS) which was rated based on therapists and physicians' clinical evaluation within one month of the study conducted.

### 2.3. Symptom Assessment

Fatigue severity was assessed using the Fatigue Scale for Motor and Cognitive Functions (FSMC). FSMC contains 20 items, and each item is scored from 1 to 5, with a higher score indicating a greater impact of fatigue on the patient's daily life. Total FSMC score represents the sum of each score from the 20 items. Anxiety and depression were assessed using the generalized anxiety disorder 7 (GAD-7) and Beck depression inventory (BDI), respectively. Real-time monitoring of total sleep time and sleep efficiency were measured using a compact wrist-worn accelerometer (Motionwatch 8, CamNtech Inc.). Participants were instructed to wear the Motionwatch on their affected wrist for four consecutive days and nights. Participants were instructed to mark their onset of sleep and wake time by pressing an indicator on the Motionwatch. “Onset of sleep” was defined as the time when the participant was in bed, had turned off the lights, and was ready to fall asleep. “Wake time” was defined as the time when the participant awoke and before they stepped out of bed. Sleep time and sleep efficiency were calculated using the Motion Ware software. Participants' data were included in the analysis only if they wore the watch and logged their sleep and wake times on at least 3 nights. Sleep time was the total number of minutes participants were asleep at night, and sleep efficiency represented the percentage of actual sleep time in reference to total time spent in bed.

### 2.4. Purification of Serum and Peripheral Blood Mononuclear Cells (PBMCs)

For serum preparation, peripheral blood was collected by venipuncture and allowed to clot for 2 hours at room temperature prior to centrifugation at 1500 rpm for 20 minutes. Serum was aliquoted and stored at −80°C prior to analysis. For purification of PBMCs, peripheral blood was collected into heparinized tubes, and PBMCs were purified by density gradient centrifugation. PBMCs were washed with phosphate-buffered saline (PBS) and suspended in RPMI 1640 medium supplemented with 10% fetal calf serum and 1% glutamine/penicillin/streptomycin.

### 2.5. Flow Cytometric Analysis of Th17 and Treg Cells

For the analysis of Th17 and Treg cells, PBMCs were suspended at a final density of 0.5 × 10^7^ cells/mL in complete culture medium (RPMI 1640 supplemented with 10% fetal calf serum and 1% glutamine/penicillin/streptomycin). Cell cultures were stimulated for 4.5 hours with phorbol 12-myristate 13-acetate (PMA, 50 ng/mL; Sigma, St. Louis, MO) and ionomycin (1 *μ*g/mL; Sigma) in the presence of Golgiplug (BD Pharmingen, San Diego, CA) at 37°C, 5% CO_2_. Th17 and Treg cells were washed and stained with human Th17/Treg phenotyping kit according to the protocol provided by the manufacturers (BD Pharmingen, San Diego, CA). Briefly, cells were fixed, permeabilized, and stained with a cocktail of fluorescent antibodies. The cocktail was composed of PerCP-Cy5.5 conjugated anti-human CD4, phycoerythrin (PE) conjugated anti-human IL-17A, and Alexa Fluor 647 conjugated anti-human Foxp3. CD4+ T cells producing IL-17A were classified as Th17 cells, whereas CD4+Foxp3+ cells were classified as Treg cells. Stained cells were analyzed with a FACSCalibur flow cytometer (BD Biosciences) and Cell Quest software (BD Biosciences).

### 2.6. Serum IL-10 Measurement

Serum levels of IL-10 were measured in duplicate using a bead based immunofluorescence assay according to the manufacturer's instructions (Millipore Inc.). Data were collected and analyzed using the Luminex-200 system version 2.3 (Luminex, Austin, TX, USA). A four- or five-parameter regression formula was used to calculate the sample concentrations from the standard curves. The threshold for detection was 0.13 pg/mL.

### 2.7. Statistical Analysis

*K*-mean cluster analysis of total FSMC scores was used to classify participants into two fatigue groups: a mild to moderate fatigue group and a severe fatigue group. Independent-sample* t*-tests were used to compare means from the two fatigue groups. IL-10 data were natural logarithmically (LN) transformed for analysis. The relationships between Th17/Treg ratio and serum IL-10, GAD-7, BDI, and mRS were analyzed using Pearson's correlation coefficient, independently. Chi-square analysis was used to examine statistically significant differences between categorical variables. *p* < 0.05 was considered statistically significant. All statistical analyses were performed using the SPSS statistical package (version 21.0).

## 3. Results

### 3.1. Participant Characteristics

**Figure 1:**
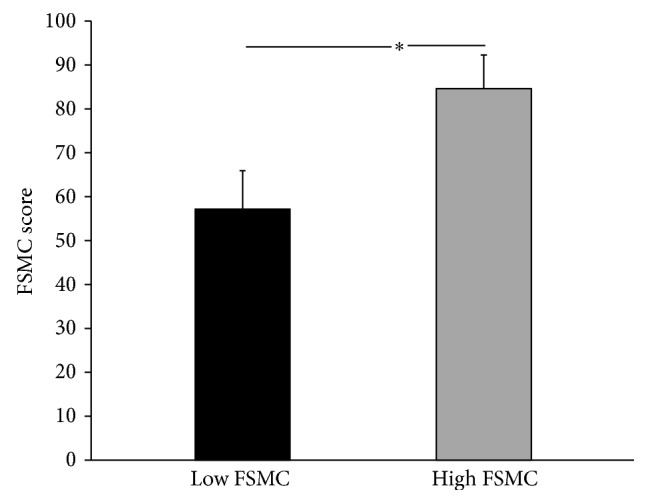
Fatigue severity classification derived from *K*-mean cluster analysis. Low FSMC represents mild to moderate PSF group (*n* = 16, mean = 57 ± 9); high FSMC represents severe PSF group (*n* = 14, mean = 84 ± 7); ^*∗*^ *p* < 0.001.

Thirty participants were recruited into the study: fourteen men and sixteen women aged between 24 and 85 years (mean age 55.6 ± 14.5 years). The mean FSMC score for the entire cohort was 69.7 ± 15.7. Participants were classified into two fatigue groups based on FSMC score by *K*-mean cluster analysis. Sixteen subjects were classified into a mild to moderate PSF group and 14 subjects were classified into a severe PSF group (57 ± 9 versus 84 ± 7, resp., *p* < 0.0001) (Figure 1). There were no significant group differences in gender, ethnicity or racial background, marital status, time since stroke, stroke type or location, stroke severity (mRS), BMI, smoking status, comorbidities, and use of immunosuppressant, steroids, or nonsteroid anti-inflammatory drugs (Table 1). There was a near significant group difference in age (*p* = 0.056); participants in the severe fatigue group were approximately 10 years younger than in the low to moderate fatigue group.

### 3.2. Relationship between Anxiety, Depression, Sleep Disturbance, and PSF

Anxiety and depression were significantly higher in the severe PSF group in comparison to the low to moderate PSF group (*p* = 0.004 and *p* = 0.001, resp.; Table 2). Mean anxiety scores were three times higher in the severe PSF group compared to those in the low to moderate fatigue group (9.1 ± 6.3 versus 3.0 ± 2.8). Similarly, depression scores in the severe PSF group were approximately twofold higher than in the mild to moderate PSF group (17.7 ± 8.8 versus 7.3 ± 5.4). There was no significant difference in sleep time or sleep efficiency between the two groups (*p* = 0.359 and *p* = 0.264, resp.; Table 2).

### 3.3. The Ratio and Percentages of Th17 and Treg in PSF

**Figure 2:**
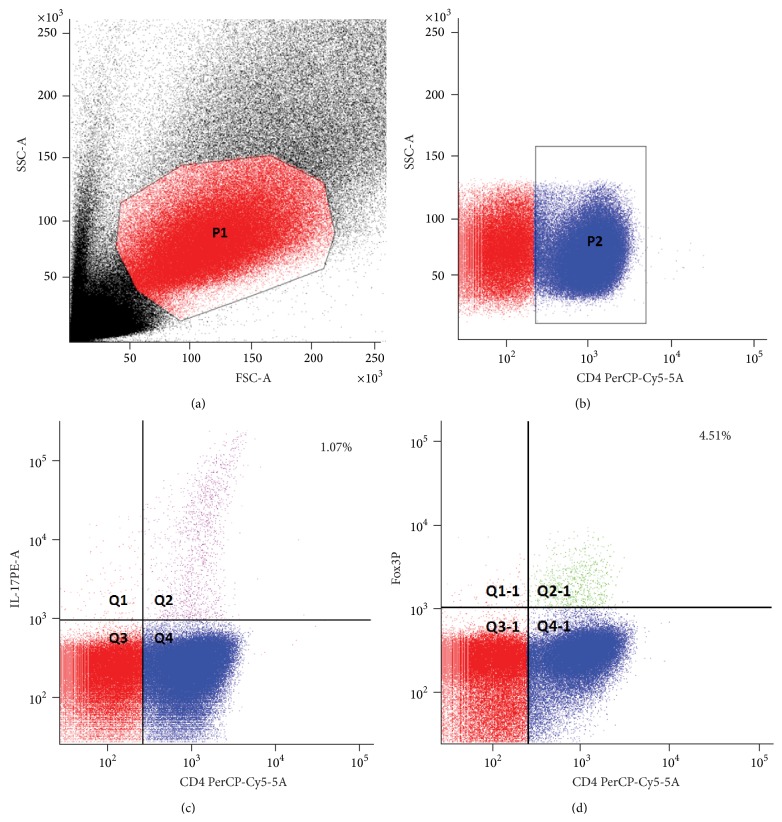
Flow cytometry analysis of Th17 and Treg cells. (a) The scatter plot shows forward and side scatter of the PBMC. (b) CD4 T cells are stained with CD4 PerCP-Cy5-5-A. (c) A percentage of Th17 cells are calculated by cells with double-positive IL17-A PE-A and CD4 PerCP-Cy5-5-A among CD4 PerCP-Cy5-5-A positive cells. (d) A percentage of Treg cells are calculated by double-positive Foxp3 APC-A and CD4 PerCP-Cy5-5-A among CD4 PerCP-Cy5-5-A cells.

The percentage of positive Th17 and Treg among CD4+ T cells was analyzed using flow cytometry (Figure 2 and Table 3). There was no significant difference in the percentage of proinflammatory Th17 cells in the severe fatigue group relative to the low-moderate fatigue group (1.07 ± 0.37 versus 0.92 ± 0.38, resp., *p* = 0.273). Similarly, there was no significant difference in the percentage of anti-inflammatory Treg cells in the severe fatigue group as compared to the mild-moderate fatigue (4.51 ± 1.67 versus 5.32 ± 1.51, resp., *p* = 0.173, Table 3). In contrast, the ratio between Th17 and Treg was significantly higher in the severe fatigue group relative to the low to moderate fatigue group (0.25 ± 0.08 versus 0.18 ± 0.08, resp., *p* = 0.035). The Th17/Treg ratio was not correlated with anxiety (*r* = 0.177, *p* = 0.375), depression (*t* = 0.059, *p* = 0.059), or stroke severity (*r* = −0.207, *p* = 0.272) (Table 4).

### 3.4. IL10 Levels Correlate with Th17/Treg Ratio

**Figure 3:**
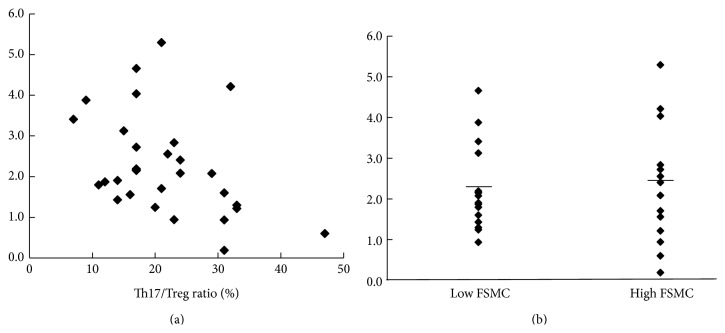
Correlation of IL-10 with T17/Treg ratio. (a) Scatter plot of correlation between Th17/Treg ratio and LN IL-10 (*r* = −0.408, *p* = 0.025). (b) Levels of LN IL-10 in low and high FSMC groups. Bars represent the mean of LN IL-10.

Although there was no significant difference in serum IL-10 levels between the low and high fatigue groups (*p* = 0.0912, Figure 3(a)), higher IL-10 levels were associated with a lower Th17/Treg ratio (*r* = −0.408, *p* = 0.025, Figure 3(b)).

## 4. Discussion

The result of the present study using flow cytometry elucidated that the ratio of Th17 to Treg in the peripheral blood was significantly elevated in the severe fatigue group in comparison to the mild and moderate fatigue group. Furthermore, the present study also demonstrated that the percentages of Th17 and Treg had no significant difference between the two fatigue groups. These results suggest that an altered balance of Th17 and Treg cells, particularly an increased ratio of Th17/Treg, not the absolute number of each of the two subsets might play a role in the development of PSF.

Previously, research had shown that proinflammatory cytokine changes of IL-1 and IL-6 associated with stroke were related to PSF [31–33]. Furthermore, another study reported that higher levels of IL-1*β* and lower levels of its antagonist IL-1ra and anti-inflammatory cytokine IL-9 in the acute phase of ischemic stroke were associated with PSF in 6 and 12 months. [33]. These findings suggest that immune-inflammatory alteration might be responsible for the pathogenesis of PSF. Notably, studies have shown that IL-1 and IL-6 play a pivotal role in mediating the differentiation of Th17 and Treg cells [14–16]. These results are in accordance with our findings that an imbalance of the Th17 and Treg ratio might be a cause of PSF. Although the Th17 and Treg ratio and function changes have been reported in stroke [24–26], there is no report on quantity and functional changes of the Th17 and Treg cells in PSF.

In the present study, we demonstrate a potential Th17/Treg homeostatic imbalance in PSF, where the percentage of Th17 and Treg does not show significant changes between the two PSF groups. It seems like a conflicting data; however, these findings are probably due to the cell-cell interaction pattern involved in inflammation development. Our findings exactly emphasized the importance of homeostasis of the Th17 and Treg interaction. A possible explanation of the Th17/Treg imbalance was implied by the genes associated with the Th17/Treg ratio. Singh et al. reported that the imbalanced Th17/Treg ratio in the late phase asthmatic response was positively correlated with almost all of the leukocyte receptor complex (LRC) on 19q13.4 which encoded the immunoglobulin super family receptors expressed on hematopoietic cells [34]. A previous study on differential profiling of LRC genes revealed that killer immunoglobulin-like receptors (KIRs, a family member of LRC) and inhibitory receptor ILT2/LIR1 were expressed in activated T cells and that KIR levels in T cells were associated with resistance to activation-induced cell death [35]. These possibly suggest a new hypothesis that the gene expression patterns of LRC might be associated with the Th17/Treg ratio and involved in the development of the PSF.

Furthermore, the present study found that Th17/Treg ratio was negatively correlated with levels of IL-10 but not correlated with anxiety, depression, or stroke severity. Results indicate that there is a descending tendency of the Th17/Treg ratio with increased levels of IL-10. The increased IL-10 might exert anti-inflammatory function to alleviate the inflammatory state of PSF. Multiple studies have shown that IL-10 plays an important role in ameliorating the neuroinflammatory response in animal stroke models [36, 37]. However, in the present study, we did not find any statistically significant difference in the levels of IL-10 between the different PSF groups, although there is a negative correlation between IL-10 and the Th17/Treg ratio in all 30 patients. A plausible explanation for this correlation is that IL-10 plays a critical role in regulating the differentiation of Th17 and Treg cells. Zheng and coworkers found that the combination of differentiated Treg cells, IL-10, TGF-*β*, and IL-2 could educate the CD4+CD25− cells to Treg cells [38]. Meanwhile, Chaudhry and coworkers found that IL-10 signaling in Treg cells is required for suppression of Th17 cell-mediated inflammation [39]. In conclusion, the relationships between IL-10 and Th17/Treg ratio in PSF indirectly support our hypothesis that an imbalance of Th17 and Treg cells is involved in the development of PSF.

The cause of fatigue is likely multifactorial, including physiological and psychological aspects. Mood disturbance has been found to be associated with PSF in several studies [1, 40]. In the present study, anxiety and depression were significantly higher in the severe fatigue group (*p* = 0.004 and *p* = 0.001, resp.). These findings were in accordance with previous research, although PSF can occur in patients who show no signs of depression [41]. Sleep disturbances are commonly related to PSF [42–44]. In this study, the sleep time and efficiency were measured by a more objective approach using Motionwatch. Nevertheless, there was no significant difference in actual sleep time and sleep efficiency between the two PSF groups. These discrepancies indirectly suggest that psychological aspects might be a basis for PSF development. In fact, PSF may have its physical origins, such as Th17/Treg cell imbalance, but perhaps may be also partially induced by psychological disorders, such as anxiety, depression, and sleep disturbance. Conversely, it is also possible that prolonged inflammation of PSF leads to altered neuronal function, which in turn causes mental and psychological disorders [45, 46]. Overall, comprehensive understanding of the putative mechanisms linked to PSF might provide a basis for better investigation of effective interventions for PSF.

A limitation of this study is the small sample size of subjects, which reduces the statistical power, precision, and validity in identifying true positives. Independent, larger sample size replication is needed as part of future studies. Another limitation is that the putative Treg cells are defined as FOXP3 positive cells in this study, as a marker for human Treg cells, but there is also transient expression of FOXP3 on small percentage of activated nonregulatory T helper cells [47]. Thus, further studies should be carried out to isolate the Treg cells based on the combination of other cell-surface markers such as CD127 and CD25 [48], because CD4+CD25+FOXP+CD127+ cells are truly representative of suppressive Treg cells.

Fatigue after stroke is a common symptom that may result from the stroke infarct but is independent of its size and location. The least controversial relationship with PSF is mood disorders, such as the anxiety and depression, although PSF can arise in their absence. Increasing evidence shows that there may be a relationship between fatigue symptoms and immune-inflammatory alteration which may affect the neural and endocrine systems. Therefore, targets at correcting immune-inflammatory alteration might provide new interventions to reduce PSF.

## 5. Conclusions

The imbalance of Th17/Treg cells is proposed to be responsible for the development and progression of PSF. Th17 and Treg cells are not present independently but to form a cross-regulation network in mediating the pathogenesis of PSF.

## Figures and Tables

**Table 1:** Participant demographic and clinical characteristics.

	Low-moderate fatigue	Severe fatigue	*p*
	(*n* = 16)	(*n* = 14)	
	Mean ± SD or *n* (%)	Mean ± SD or *n* (%)	
Age (years)	60.2 ± 16.8	50.4 ± 9.4	0.056
Male gender	9 (56.3)	7 (50.0)	0.732
Ethnicity			
Hispanic/Latino	2 (12.5)	2 (14.3)	0.648
Non-Hispanic/Latino	14 (87.5)	12 (85.7)	
Racial background			
Non-Caucasian or Non-White	5 (31.3)	6 (42.3)	0.228
Caucasian or White	11 (68.7)	8 (57.7)	
Marital status			
Married or widowed	10 (62.5)	11 (78.6)	0.440
Single or divorced	6 (37.5)	3 (21.4)	
Time since stroke (months)	43.7 ± 56.2	26.8 ± 28.8	0.323
Stroke side			
Right	8 (50.0)	5 (35.7)	0.431
Left	8 (50.0)	9 (64.3)	
Stroke type			
Ischemic	13 (81.3)	7 (50.0)	0.122
Hemorrhagic	3 (18.7)	7 (50.0)	
Stroke location			
Supratentorial	15 (93.7)	12 (85.7)	0.586
Infratentorial	1 (6.3)	2 (14.3)	
Stroke severity			
mRS	2.6 ± 0.8	2.4 ± 0.7	0.359
BMI, kg/m^2^	27.3 ± 4.4	27.5 ± 5.3	0.935
Current smoker	2 (12.5)	2 (14.3)	0.648
Comorbidities			
Hypertension	6 (37.5)	10 (71.4)	0.063
Diabetes	2 (12.5)	1 (7.1)	0.552
AF	2 (12.5)	0 (0.0)	0.485
CAD	2 (12.5)	0 (0.0)	0.485
Thyroid disease	0 (0.0)	0 (0.0)	—
Drug treatment			
Immunosuppressant	0 (0.0)	0 (0.0)	
Prednisolone	1 (6.3)	1 (7.1)	0.724
NSAIDs	0 (0)	2 (14.3)	0.209

Continuous variables are presented as mean ± standard deviation, whereas categorical variables are expressed as counts and percentages. mRS is modified Rankin Scale; BMI indicates body mass index; AF is atrial fibrillation; CAD is coronary artery disease; NSAIDs is nonsteroidal anti-inflammatories.

**Table 2:** Anxiety, depression, and sleep disturbance in the PSF patients.

	Low-moderate fatigue	Severe fatigue	*p *
	(*n* = 16)	(*n* = 14)	
GAD-7 scores	3.0 ± 2.8	9.1 ± 6.3	0.004^*∗*^
BDI scores	7.3 ± 5.4	17.7 ± 8.8	0.001^*∗*^
Actual sleep time	438.3 ± 86.9	469.4 ± 85.6^*∗∗*^	0.359
Sleep efficiency	86.9 ± 8.6	84.8 ± 8.8^*∗∗*^	0.264

Values are expressed as mean ± standard deviation.

The differences between low and high FSMC groups were determined by *K*-mean cluster analysis.

^*∗*^ *p* values less than 0.05 were considered statistically significant.

^*∗∗*^2 missing data for these sleep variables in the high PSF group; that is, *n* = 12.

**Table 3:** The percentages and ratio of Th17 and Treg cell in PSF patients.

T cell	Low-moderate fatigue	Severe fatigue	*p*
	(*n* = 16)	(*n* = 14)	
Th17%	0.92 ± 0.38	1.07 ± 0.37	0.273
Treg%	5.32 ± 1.51	4.51 ± 1.67	0.173
Th17/Treg	0.18 ± 0.08	0.25 ± 0.08	0.035^*∗*^

Values are expressed as the mean ± standard deviation. The low and high FSMC groups were generated by *K*-mean cluster analysis.

^*∗*^ *p* values less than 0.05 were considered statistically significant.

**Table 4:** Correlation of Th17/Treg ratio with anxiety, depression, and stroke severity.

Variables	Th17/Treg ratio	
	*r*	*p*
GAD-7 scores	0.177	0.350
BDI	0.059	0.059
mRS	−0.207	0.272

Anxiety was measured by GAD-7 scores. Depression was measured by BDI. Stroke severity was measured by mRS. The correlation between Th17/Treg ratio and GAD-7, BDI, and mRS was analysed using Pearson's correlation coefficient.
